# A high-quality RNA-yielding protocol for laser capture microdissection of transplanted stem cell-derived Islets of Langerhans

**DOI:** 10.1007/s00592-026-02654-z

**Published:** 2026-02-09

**Authors:** Daniel Norman, Joey Lau

**Affiliations:** Present Address: Department of Medical Cell Biology, Uppsala University, 751 23 Uppsala, Sweden

**Keywords:** Laser capture microdissection, Stem cell-derived islets, RNA quality, Transplantation, Type 1 diabetes

## Abstract

**Background:**

Laser capture microdissection (LCM) followed by RNA-sequencing is a powerful, widely applicable tool to analyze the transcriptome in regions of a tissue. Protocols for LCM of transplanted islets of Langerhans, particularly stem cell-derived islets (SC-islets) that have evaluated RNA quality, are lacking. This study demonstrates a robust protocol for LCM of SC-islets in multiple organ sites, generating high quality RNA.

**Method:**

SC-islets were transplanted to five organ sites in immunodeficient NOG-mice. Graft-containing organs were then sectioned, fixed in 75% ethanol, stained with the alcohol-based stain cresyl violet, and dehydrated before performing LCM. RNA was then extracted, and quality control was performed.

**Results:**

High RIN scores (RNA Integrity Number) were obtained from all organ sites, with the pancreas showing the most robust results, despite its known challenges due to high RNase content. Conversely, organs with small or dispersed grafts, such as the liver and omentum, exhibited lower RIN scores. This is likely due to the size of the dissected area correlating positively with RIN scores, potentially due to a more time-consuming LCM in these sites.

**Conclusion:**

Using this novel protocol, high-quality RNA from transplanted SC-islets can be obtained. Smaller and spread-out grafts pose a challenge in obtaining higher quality RNA, although possible.

**Supplementary Information:**

The online version contains supplementary material available at 10.1007/s00592-026-02654-z.

## Introduction

Laser capture microdissection (LCM) is used to dissect specific regions of a tissue to subsequently extract RNA or protein [1, 2]. Downstream possibilities are e.g., qPCR and RNA sequencing, making LCM a powerful tool to evaluate transcriptomics of specific cell populations.

Achieving high-quality RNA from LCM can be challenging, and protocols must take into consideration some major challenges, such as small obtained quantities of RNA, room temperature and humidity during LCM and special tissue handling regarding freezing, fixation, storage, and staining. Most steps risk lowering RNA quality [3]. There is also a limitation in what tissues can be used since formalin fixed tissues render poorer RNA quality [4]. This limits the use of samples from biobanks.

Despite being an established method for over twenty years, optimization of protocols for LCM in different settings is still ongoing. In the case of LCM of native islets of Langerhans, several protocols exist [5–10], with some evaluating RNA quality in terms of RIN value. However, protocols that have evaluated the RNA quality of transplanted islets, particularly human stem cell-derived islets (SC-islets), are, to our knowledge, nonexistent. Therefore, we evaluated our LCM-protocol for transplanted SC-islets in multiple sites (pancreas, liver, kidney capsule, omentum, and muscle), which is based on and modified from previous publications. This protocol also enables RNA-sequencing of islets transplanted to organs where the islets are non-retrievable surgically, such as the liver.

## Method

### Differentiation of SC-islets

Human embryonic stem cells (hESC) from the H1 cell line (Wicell^®^, Madison, WI, USA) were propagated in mTeSR-Plus medium (#100–0274/100–0275, STEMCELL Technologies) on human recombinant Laminin 521 (LN521, BioLamina) in a CO_2_ incubator at 37 °C, 5% CO_2_, and 100% humidity. H1 cells were seeded on Laminin 521-coated plates at a density of 16 million cells/10 cm dish or two million cells/3.5 cm dish in mTeSR-Plus medium with the addition of 10 µM ROCK inhibitor Y-27,632 (#72304, STEMCELL Technologies). A seven-stage differentiation protocol was used to differentiate the H1 cells into stem cell-derived islets as previously published [11], incorporating specific modifications as described previously [12]. At stage seven and 2–7 weeks, SC-islets were transplanted.

## Animals

NOD.Cg-Prkdc^scid^I12rg^tm1Sug^ (NOG) mice (Taconic M&B, Ejby, Denmark; female and male mice, 20.1 ± 2.3 g, aged 7–20 weeks) were used as recipients for the transplantation of SC-islets. All experiments were approved by the Animal Ethics Committee in Uppsala, Sweden.

## Transplantation of SC-islets

Transplantation of 700–800 islet equivalents of SC-islets was carried out as previously described [13–15]. Briefly, SC-islets were packed in a PE-50 tubing and implanted beneath the renal capsule or packed in a braking pipette and injected into a surgically created omental pouch. For the implantation organs liver, pancreas and striated muscle, the SC-islets were injected through a butterfly needle (25-gauge) into the portal vein, into the splenic part of the pancreas or into the abdominal muscle, respectively. At harvest, grafts were embedded in OCT and flash frozen in isopentane surrounded by liquid nitrogen and stored at -80 °C.

## Material list for LCM

LCM-microscope (Leica LMD6000 B microscope, Leica Microsystems).

Cryostat (CryoStar NX70, Thermo Fisher Scientific, Waltham, MA, USA).

Tissue-Tek^®^ O.C.T. Compound (Sakura Finetek USA, Inc., Torrance, California, USA).

Cresyl violet (Cresyl Violet acetate, # 10501481, Fisher Scientific, Göteborg, Sweden).

Thermo Scientific™ RiboLock RNase Inhibitor (40 U/µL; # 10389109, Fisher Scientific, Göteborg, Sweden).

LCM FrameSlides (MDE5P80WFK, FrameSlides, PPS-membrane 4.0 μm, RNase-free, MicroDissect GmbH, Herborn, Germany).

Leica frame support for easier mounting.

Microscope slide containers (5-piece) for dehydration; FrameSlides are packed in these.

Cleanable plastic slide box for staining.

Tweezer for moving slides.

RNase-free 500 µl tubes for sample collection.

Ethanol diluted with RNase-free water (50, 75, 95 and 100%).

Xylene.

RNase cleaning solution and/or RNA and DNA cleaning UV-box.

Charged glass slides.

Qiagen RNeasy plus micro kit (cat. No. 74034, Qiagen, Germantown, USA).

Dithiothreitol (cat. No. R0861, ThermoFisher, Waltham, Massachusetts, USA) for adding to the lysis buffer.

## General remarks

Apply basic RNase-free practices during the whole protocol, using gloves and RNase-free equipment when possible and work in clean, dust free spaces.

This protocol is carried out in one session to avoid unnecessary freezing-thawing, although other protocols pause after sectioning (see below). Dehydrating sections before LCM and avoiding water-based solutions whenever possible – e.g. when staining – is key to preserving RNA [16, 17]. Many protocols use a rehydration step after fixation to use a water-based staining, which is detrimental to RNA; therefore, this protocol uses an alcohol-based staining with cresyl violet, adopted from Bevilacqua et al. [18]. Cresyl violet also provides an excellent contrast between SC-islet grafts and organ site tissue, facilitating identification and LCM.

The protocol is adjusted to handling 1–5 FrameSlides, but more can be handled simultaneously by doubling the number of containers. However, handling too many slides can affect staining times and prolong LCM, and therefore possibly lower RNA quality.

The time required to carry out the protocol, from sectioning to a lysed sample in the freezer, is roughly five hours. The most time-consuming factor is finding the transplant while cryosectioning. Secondly, LCM time varies depending on transplant size and distribution in the organ.

### Freezing tissue

Organs containing transplants are excised as rapidly as possible, instantly embedded in OCT and frozen in isopentane chilled with liquid nitrogen. We followed a protocol by Dewan and Loomis at NYU Langone medical center [19]. Performing this step quickly is crucial to limit endogenous RNase activity.

## General preparations

Cresyl violet (1% w/v) was prepared by adding 0.2 g cresyl violet to 20 ml 75% ethanol and leaving the mixture on a shaker for 1 h. The cresyl violet solution was then filtered through a 0.8 μm pore filter, which required multiple filters due to clogging. The solution was then split into Eppendorf tubes to avoid contamination and evaporation of ethanol.

The frame support, cryostat sample holder, microscope slide containers, staining box, glass slides for histology, and tweezers were cleaned by wiping with 100% ethanol and then UV-treated for 40 min (this can be done in the cryostat if available or, as in this case, on a separate workbench with UV-light).

Lysis buffer for sample collection was prepared according to the instructions for LCM-samples in the manual for Qiagen RNeasy Micro Plus Kit, using dithiothreitol (DTT) instead of beta-mercaptoethanol,* which is both smelly and highly toxic.*

## Cryosectioning

### Preparations

Absolute ethanol was diluted with RNase-free water to use for storage of FrameSlides in the cryostat and dehydration preceding staining; 20 ml per microscope slide container and 1 × 50%, 1 × 75%, 2 × 95% and 2 × 100% (% of ethanol to RNase-free water). The microscope slide container with 75% ethanol was placed in the cryostat. Two additional containers were filled with 20 ml of xylene each. Note that some types of plastic are melted by xylene. The cryostat and a new sectioning blade were cleaned with 100% ethanol. If available, UV-treat the cryostat. A microscope slide container was filled with 3–4 cm of cresyl violet (prepared above) and a beaker with water for rinsing the cresyl violet-stained slide was prepared; they were used to localize the graft while sectioning. A container with new FrameSlides was stored in the cryostat until use, since keeping them cold improves RNA yield [18]. The FrameSlide support was kept at room temperature since sections must melt slightly to adhere. The tissue block to be sectioned was equilibrated to cryostat temperature (which varies with different tissues) for 15 min before cryosectioning.

### Cryosectioning

Cryosectioning was performed with a 10 μm thickness. Transplants were localized by staining a section on a glass slide by dipping it in cresyl violet for approximately five seconds at room temperature, then dipping it clean in water and blotting it on a paper towel. The graft was then localized using a regular light microscope. When the transplant was found, sections were put on FrameSlides using the warm frame support and cold FrameSlides. Multiple levels of grafts were included to ensure the inclusion of heterogeneous parts. E.g., since regular glass slides were obtained for histology, every fifth section was mounted on a FrameSlide and the rest on glass slides for histology.

Every time a FrameSlide was filled, it was placed in the slide box, which was filled with cold 75% ethanol, inside the cryostat. The number of filled FrameSlides needs to be adjusted to the application in question. For example, liver grafts required around five FrameSlides to yield enough RNA, due to their small size.

When the cryosectioning was completed and the needed number of LCM-membranes were stored in 75% ethanol in the cryostat, staining was carried out within an hour.

Sections for histology were air dried for 30 min and collected during later incubation steps in the subsequent protocol, and stored at -80 °C.

Some protocols pause here by freezing FrameSlides in Falcon tubes instead of placing them in 75% ethanol. However, since storage at -80 °C has been shown to yield lower amounts of RNA [18, 20], this protocol continues the same day – even though refreezing sections with good results seems possible [3]. *Storing FrameSlides in 75% ethanol preserves RNA quality*, and could, according to another study, even be stored until the next day [18]; in this protocol, we, however, continued within an hour. Additionally, using 100% instead of 75% ethanol could theoretically reduce RNase activity more due to less water allowing RNase activity,* but it does not dissolve OCT from the section*, impairing subsequent staining.

### Staining and dehydration

Staining is carried out in a clean, dust free fume hood. Use a slide box that only touches the edges of the FrameSlide, not the membrane itself. Incubations in ethanol and xylene are carried out in the prepared microscope slide containers, while cresyl violet is added to slides laying flat in the cleaned staining box. Sections on frame slides were stained and dehydrated in this sequence using the microscope slide containers prepared above: EtOH 50% – 20 s; cresyl violet – 30 s; EtOH 50% – 5 s; EtOH 75% – 30 s; EtOH 95% − 1 min; EtOH 95% − 1 min; EtOH 100% – 1 min; EtOH 100% – 1 min; xylene – 5 min; xylene – 5 min. Sections were then air dried until the xylene had evaporated, tilting the box containing FrameSlides and blotting the edges with clean paper to speed up the process.

### Laser capture microdissection

During the two long xylene incubations during staining, preparations for LCM can be made to save time.

### Preparations

Microscope surfaces were wiped, particularly the sample holder, with 100% ethanol. The sample holder was prepared by adding 50 µl lysis buffer (from the Qiagen RNeasy Plus Micro Kit) to the cap of an inserted Eppendorf tube. 40 U/µl RNase-inhibitor (Thermo Scientific™ RiboLock RNase Inhibitor) was added to the tube cap for RNA-protection during dissection and storage. The need for an RNase-inhibitor can be questioned; see discussion.

### Dissection

Dissection is preferably completed as fast as possible. However, since the sections are dehydrated in xylene, only small amounts of water can be reabsorbed to activate RNases. It is a good idea to orient oneself morphologically in the different grafts using light microscopy and/or fluorescent staining with insulin and glucagon on separate slides, before performing the LCM. The LCM microscope we use,* Leica LMD6000 B*, cuts whole pieces of tissue. Thus, we mark several independent areas, cut in whole pieces, and collect them in the same tube.

Settings for cutting were calibrated for each section. This varied with almost every slide, which makes the calibration vital. The transplants were marked and the appropriate sample collector (prefilled with lysis buffer) was chosen, dissecting as much of the grafts as possible. In this protocol, aiming for at least 4 mm^*2*^ dissected tissue was sufficient to yield good-quality RNA samples.

**Fig. 1:**
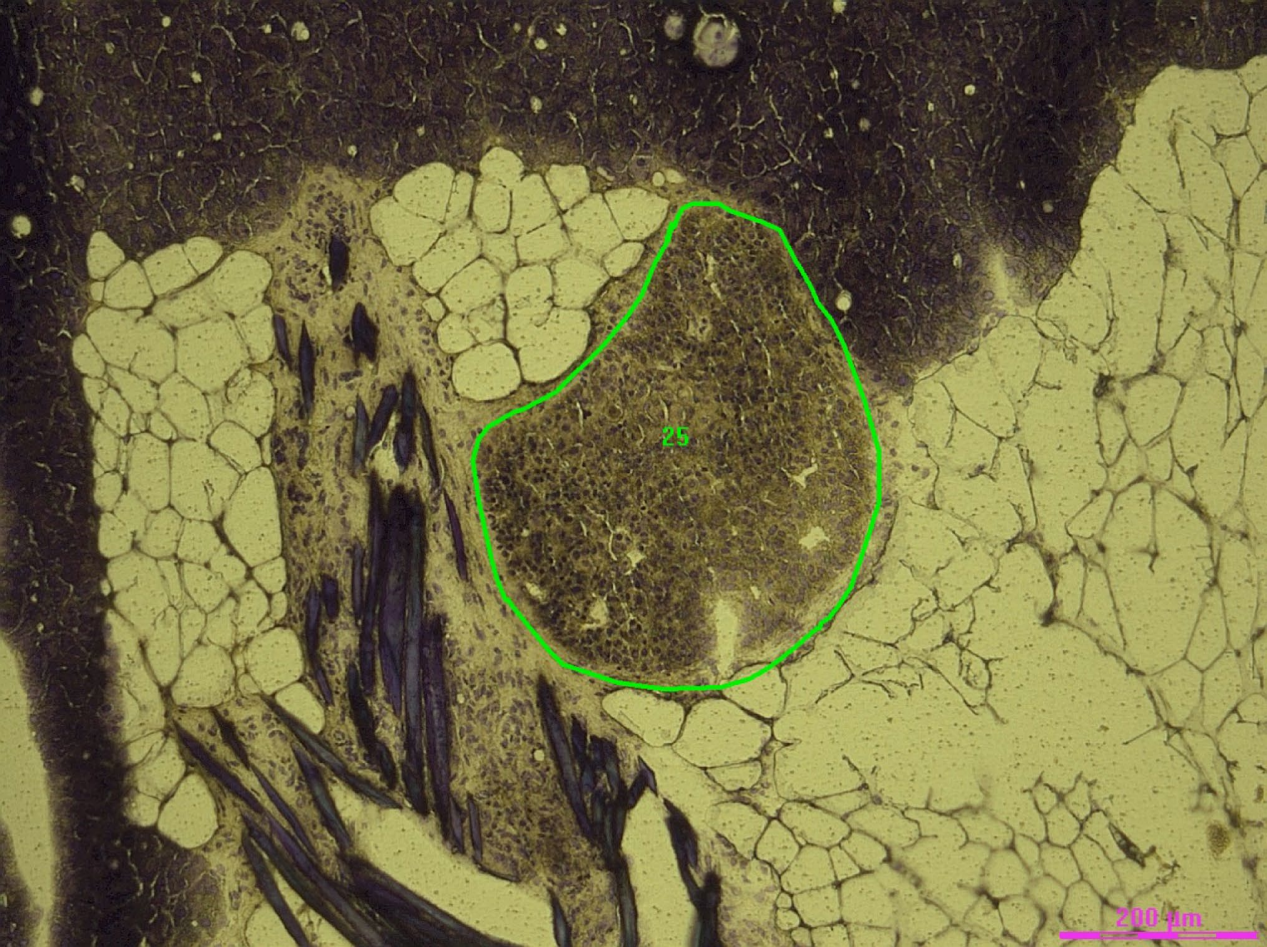
Example of a graft marked for dissection: a stem cell-derived islet graft in the omentum, close to the pancreas and the suture (left of graft) holding the omental graft pouch together. Scale bar 200 μm

**Fig. 2:**
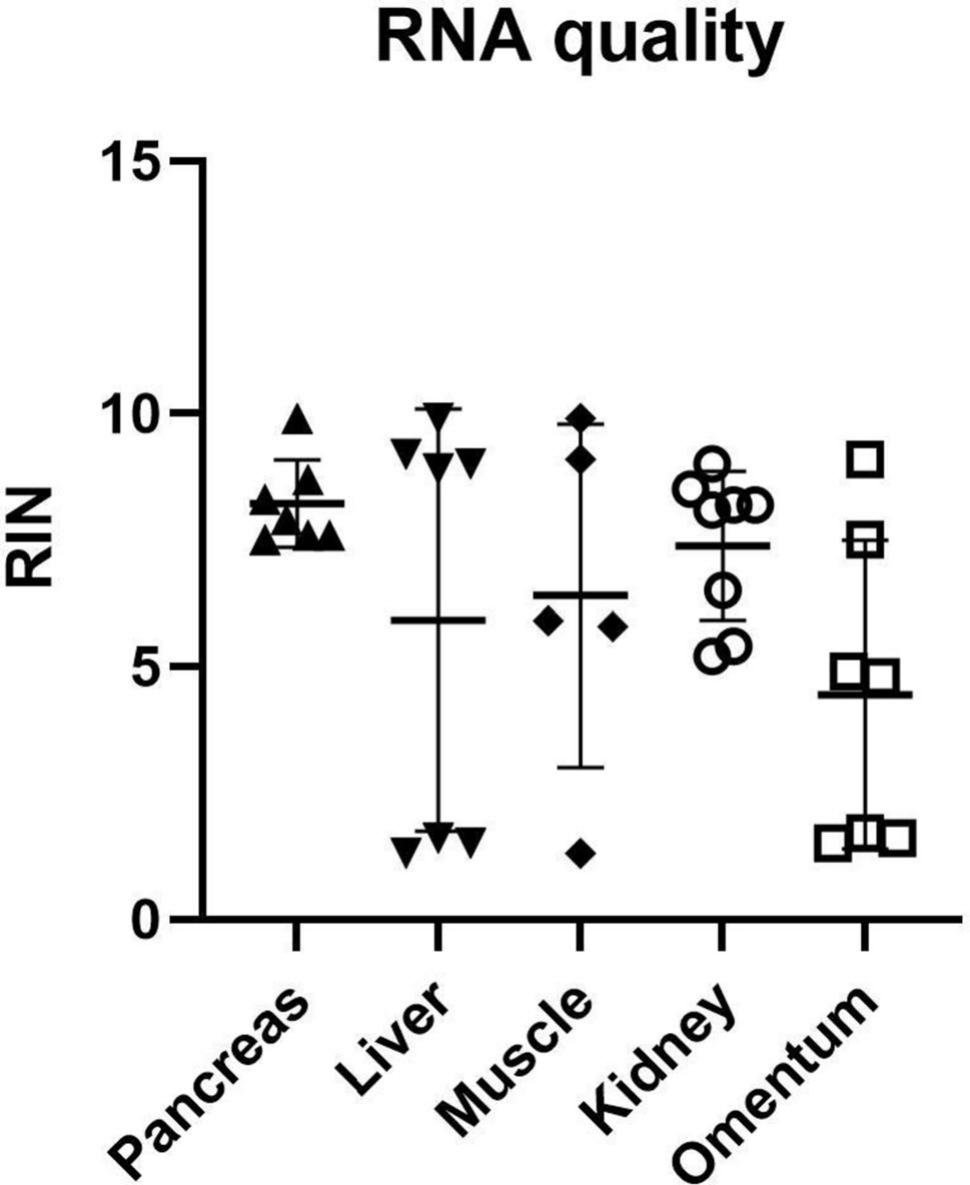
RNA Integrity Number (RIN) values for each transplantation site are expressed as mean ± SD

After dissection, the sample collector was removed and centrifuged. Lysis buffer with DTT was added to the samples, bringing the total volume to 350 µl (using the Qiagen RNeasy Micro Plus Kit). The sample was then vortexed for 30 s and stored at -80 °C – where they are stable for months – until extraction (Figs. 1 and 2).

### Extraction and quality control

Extraction was performed with Qiagen RNeasy Micro Plus according to the manufacturer’s instructions. Analysis of RNA quality was performed using the Agilent 5400 Fragment Analyzer (Agilent, Santa Clara, CA, United States).

## Results

**Fig. 3:**
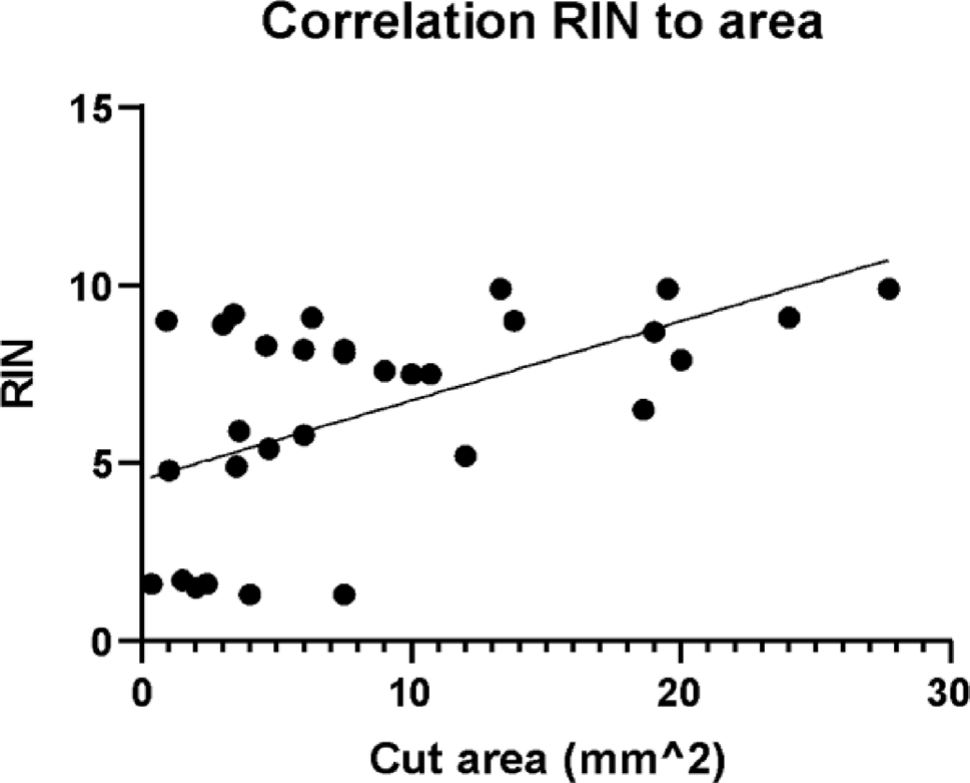
Correlation between RNA Integrity Number (RIN) value and dissected area for each sample regardless of sample group. Slope 0.23, R squared 0.3

For all transplant sites, RNA with high RIN value was obtainable: pancreas (RIN 7.5–9.9; mean 8.2), liver (RIN 1.3–9.9; mean 5.9), muscle (RIN 1.3–9.1; mean 6.4), kidney capsule (RIN 5.2–9; mean 7.4) and omentum (RIN 1.5–9.1; mean 4.4). Individual sample values for ng of RNA, dissected area and RIN-value are seen in Table 1 below. Despite all sites having individual RIN values > 9, liver and omentum had a particularly low mean value. Correlating the dissected area to the RIN value shows that the bigger the dissected area, the higher the RIN, as seen in Fig. 3.

**Table 1:** Individual values of ng RNA obtained, area dissected and RIN-value for the different transplantation sites

Pancreas			
Total amount RNA (ng)	mm^2^ dissected	ng/mm^2^	Integrity value
25.47	27.7	0.92	9.9
29.91	19.0	1.57	8.7
25.10	4.6	5.46	8.3
28.84	14.3	2.48	8.2
57.84	20.0	2.89	7.9
30.36	9.0	3.37	7.6
29.46	10.7	2.75	7.5
3.75	9.0	0.42	7.6
**Liver**			
**Total amount RNA (ng)**	**mm**^**2**^ **dissected**	**ng/mm** ^**2**^	**Integrity value**
15.17	13.3	1.14	9.9
3.72	3.4	1.09	9.2
2.34	0.9	2.60	9.0
2.69	3.0	0.90	8.9
5.78	4.1	1.87	5.9
0.81	2.4	0.34	1.6
12.33	2.0	6.17	1.5
3.42	4.0	0.85	1.3
**Abdominal muscle**			
**Total amount RNA (ng)**	**mm**^**2**^ **dissected**	**ng/mm** ^**2**^	**Integrity value**
9.86	19.5	0.51	9.9
45.55	24.0	1.90	9.1
18.85	12.1	1.54	6.4
0.67	3.6	0.19	5.9
0.86	6.0	0.14	5.8
37.31	7.5	4.97	1.3
**Kidney**			
**Total amount RNA (ng)**	**mm**^**2**^ **dissected**	**ng/mm** ^**2**^	**Integrity value**
31.36	13.8	2.27	9.0
23.24	6.0	3.87	8.2
18.81	7.5	2.51	8.2
10.07	7.5	1.34	8.1
19.05	10.0	1.96	7.2
21.38	18.6	1.15	6.5
1.72	4.7	0.36	5.4
26.74	12.0	2.23	5.2
**Omentum**			
**Total amount RNA (ng)**	**mm**^**2**^ **dissected**	**ng/mm** ^**2**^	**Integrity value**
9.74	6.3	1.55	9.1
2.26	10	0.23	7.5
0.82	3.5	0.23	4.9
31.57	1.0	31.57	4.8
8.31	3.5	8.73	4.4
0.96	1.5	0.64	1.7
8.70	0.35	24.87	1.6
4.10	2.0	2.50	1.5

## Discussion

High quality RIN samples (> RIN 9) were obtainable in all graft sites. However, mean RIN values were lower than that. In a regular project, some samples are naturally discarded after quality control. Thus, it is fully possible to obtain a good number of observations with high RIN-value by choosing an appropriate sample size.

Most of the low RIN values also had a smaller dissected area, as seen in the correlation between the dissected area and RIN. This likely affects sites with smaller grafts negatively, such as omentum and liver, which is seen in the broader distribution of RIN-values in Fig. 2. The main reason is presumably that dissection of many small grafts is more time consuming, resulting in longer time at room temperature.

Surprisingly, the pancreas, generally considered challenging for RNAextraction due to a high RNase content, had the most robust RNA quality. One reason for this could be the big, clustered transplants allowing for easier dissection with larger amount of RNA.

Regarding the use of RNase-inhibitors in LCM-protocols, the value is debatable, and likely depends on how the rest of the protocol is designed. Protocols avoiding water-based stainings such as hematoxylin, and that dehydrate samples thoroughly before LCM, can function well without an RNase-inhibitor [3, 8, 9, 17]. Thus, RNase-inhibitors are probably not necessary, albeit not harmful. One exception is immuno-LCM, where sections are antibody labeled, thus requiring water-based solutions. In these protocols, using an RNase-inhibitor during staining clearly helps [16, 21, 22], since RNA quantities quickly decline in water [16].

In summary, although small and spread-out grafts pose a challenge in obtaining high-quality RNA, our protocol works well for extracting RNA from transplanted SC-islets in various sites, and is therefore, likely applicable to regular transplanted islets of Langerhans.

## Supplementary Information

Below is the link to the electronic supplementary material.

Supplementary Material 1
