# Supplementary material for: A high-quality RNA-yielding protocol for laser capture microdissection of transplanted stem cell-derived Islets of Langerhans
Source: Acta Diabetol. 2026 Feb 9;63(5):801–7. doi: 10.1007/s00592-026-02654-z (PMC13219057; doi:10.1007/s00592-026-02654-z)

Conflict of Interest Form

Conflict of Interest Policy:

Authors are required to disclose commercial or similar relationships to products or companies mentioned in or related to the subject matter of the article being submitted. Affiliations of authors should include corporate appointments relating to or in connection with products or companies mentioned in the article, or otherwise bearing on the subject matter thereof. Sources of funding for the article should be included in the acknowledgments. Other pertinent financial relationships, such as consultancies, stock ownership, or other equity interests or patent-licensing arrangements, should be disclosed in the cover letter to the Editor-in-Chief, on a separate conflict of interest page in the manuscript (see below for examples of how to format the conflict of interest page in your manuscript) and on the conflict of interest form accompanying the article at the time of submission. The conflict of interest form, which is available at: http://www.springer.com/774, should be signed, scanned and submitted through Editorial Manager. The conflicts of interest disclosed on the conflict of interest form should be the same as those disclosed on the conflict of interest page in the manuscript. Questions about this policy should be directed to the Editor-in-Chief.
Please note: When considered necessary, the raw data of a manuscript will be requested to be submitted and examined by a third-party.

Examples:

The conflict of interest page should take the form of a statement as shown in the following examples.

- Dr. YYYYY serves as a consultant for Company X.
- Dr. XXXXX is an employee of Company Y.
- Dr. XXXXX owns stock in Company Z.
- All other authors have no conflicts of interest.
- If no author has a conflict, the statement should read “All authors have no conflicts of interest.”

If there is a conflict of interest, check the appropriate “Yes” box below and provide details. If the listed relationship does not apply to you or a family member, check the appropriate “No” box.

Category	No	Yes	If yes, give names of authors and entities.
Consultant	X		
Employment	X		
Stock Ownership	X		
Other equity interests or patent-licensing arrangements	X		

Date: 22.03.2025 First author’s signature: 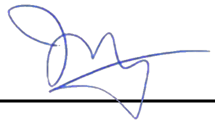

Supplement: Supplementary file 1 — Supplementary Material 1 [file 592_2026_2654_MOESM1_ESM.pdf]
